# The Evaluation of the Neuroprotective Effect of a Single High-Dose Vitamin D_3_ in Patients with Moderate Ischemic Stroke

**DOI:** 10.1155/2022/8955660

**Published:** 2022-01-17

**Authors:** Omid Hesami, Setare Iranshahi, Shima Zareh Shahamati, Mohammad Sistanizd, Elham Pourheidar, Rezvan Hassanpour

**Affiliations:** ^1^Department of Neurology, Imam Hossein Medical and Educational Center, Shahid Beheshti University of Medical Sciences, Tehran, Iran; ^2^Student Research Committee, Department of Clinical Pharmacy, Faculty of Pharmacy, Shahid Beheshti University of Medical, Tehran, Iran; ^3^Department of Clinical Pharmacy, Faculty of Pharmacy, Shahid Beheshti University of Medical Sciences, Tehran, Iran; ^4^Prevention of Cardiovascular Disease Research Center, Imam Hossein Medical and Educational Center, Shahid Beheshti University of Medical Sciences, Tehran, Iran

## Abstract

**Introduction:**

Vitamin D insufficiency is highly prevalent and is a negative predictor for survival in ischemic stroke patients. We evaluated the effect of a high dose of vitamin D_3_ on the Neuron-Specific Enolase (NSE) level, National Institute of Health Stroke Scale (NIHSS), and Barthel Index (BI) scoring system in moderate ischemic stroke patients.

**Methods:**

This prospective, double-blind, randomized clinical trial (RCT) study was conducted from April 2020 to March 2021. Patients with moderate ischemic stroke (NIHSS 5 to 15) who had vitamin D deficiency (serum 25-OH vitamin D ≤30 ng/mL) were recruited and randomized into intervention and control groups. Subjects in the intervention group received a single dose, intramuscular (IM) injection of 600000 international unit (IU) vitamin D_3_, in addition to the standard treatment. NSE level and NIHSS were evaluated at baseline and 48 hours after the intervention. The BI was monitored three months after discharge.

**Results:**

During the study period, 570 patients were assessed; finally, forty-one patients completed the study. Except for the age which was higher in the control group (*p* = 0.04), there were no statistically significant differences in other baseline characteristics between the two groups. After 48 hours, the NIHSS score was significantly lower in the intervention group (median 8 vs. 6.5, *p* = 0.008 in the control and intervention groups, respectively), but there was no significant difference in the NSE level (*p* = 0.80). Three months after discharge, the BI was significantly higher in the intervention group (median 8 vs. 9, *p* = 0.03 in the control and intervention groups, respectively).

**Conclusions:**

Administration of a single 600000 IU of vitamin D_3_ could have neuroprotective effects in patients with moderate ischemic stroke, according to its significantly positive effects on functional clinical outcomes (NIHSS and BI), but this effect on the biomarker related to neural damage (NSE) was not significant.

## 1. Introduction

Vitamin D insufficiency is highly prevalent in acute ischemic stroke patients compared with the patients without stroke [1]. Also, there is an association between the severity of vitamin D deficiency and ischemic infarct volumes, functional outcomes, and stroke recurrence. Therefore, vitamin D deficiency could be considered as a negative predictor for survival in patients with ischemic stroke [1–4].

Several mechanisms have been proposed for the neuroprotective properties of vitamin D [5]. Vitamin D promotes the expression of insulin-like growth factor 1 (IGF-1) which has neuroprotection capabilities [6]. Also, it has been suggested that vitamin D has antithrombotic and vasodilatory effects which, therefore, improve the blood flow of neurons [7]. Vitamin D, as an antioxidant, with inhibition of reactive oxygen can prevent blood-brain barrier (BBB) dysfunction after an ischemic stroke [5, 8].

Vitamin D supplementation is suggested to reduce neurological, psychological, and musculoskeletal complications in stroke patients. Poststroke patients would benefit from the antidepressive and anticonvulsant effects of vitamin D [9, 10]. Also, vitamin D supplementation could improve muscle strength in poststroke patients with hemiplegia and improve their motor functions [11].

Nowadays, besides stroke severity and prognosis scales, serum biomarkers are investigated for diagnosis and outcome prediction in ischemic stroke patients [12]. Neuron-Specific Enolase (NSE), an enzyme released after neuronal damage, has been studied as a marker for brain injury including ischemic stroke [13], and NSE level correlates with a patient's clinical deficits and infarct volume [14, 15].

Most randomized controlled trials (RCTs) directly investigated the effects of the oral forms of vitamin D in stroke patients [5], while intramuscular (IM) injection of single high doses of vitamin D can increase serum 25-OH vitamin D level rapidly and safely and also could improve patients' balance performance [16]. Few studies examined the effect of single high doses of vitamin D on functional outcome scales and did not evaluate the serum biomarkers in patients with ischemic stroke [17, 18].

Therefore, with this knowledge gap in the background, we designed this study to evaluate the effect of a high dose of vitamin D_3_ on the NSE level as a neuromarker, National Institute of Health Stroke Scale (NIHSS), and Barthel Index (BI) scoring system as the functional outcomes in patients with moderate ischemic stroke.

## 2. Methods

### 2.1. Settings

The present prospective, double-blind, randomized clinical trial (RCT) study was conducted in the neurology ward of Imam Hossein Medical Center, affiliated with Shahid Beheshti University of Medical Sciences (SBMU) in Tehran, Iran, from April 2020 to March 2021. This study has been approved by the Institutional Review Boards of the Ethics Committee of SBMU (IR.SBMU.PHARMACY.REC.1399.213). Also, the study protocol was registered, reviewed, and approved by the Iranian Registry of Clinical Trials (IRCT), with the registry number of IRCT20120703010178N24.

### 2.2. Study Population

Adult patients suffering from moderate ischemic stroke, NIHSS score 5 to 15, admitted to the neurology ward during the last 24 hours with vitamin D deficiency (serum 25-OH vitamin D ≤30 ng/mL) were included. Patients with a history of acute or chronic renal (creatinine clearance (CrCl) (<30 mL/min)/1.73 m^2^) and liver failure were excluded from the study.

### 2.3. Interventions

Written informed consent was obtained from each subject before enrollment in the study. Included patients were randomized into two groups of intervention and control. Randomization was done by simple randomization method, using series of random numbers generated by randomize (RND) command of Excel software. All patients were managed according to the standard treatment protocol based on the AHA/ASA (American Heart Association/American Stroke Association) guidelines [19]. The intervention group, in addition to the standard treatment, received a single dose, IM injection of 600000 international unit (IU) vitamin D_3_ (Daroupakhsh Co. Ltd., Tehran, Iran). Subjects in the intervention group were kept blinded to the study intervention.

### 2.4. Assessments

The baseline data consist of age, sex, serum 25-OH vitamin D level, and hospital length of stay and were recorded for all patients. To evaluate NSE level, venous blood samples were collected at baseline (NSE 0) and 48 hours after recruitment (NSE 1). The serum was separated by centrifuged (at 2000 rotations per minute (rpm) for 10 minutes) and immediately stored at -80 °C. NSE levels were measured by using human Enzyme-Linked Immunosorbent Assay (ELISA) kits (CanAg Diagnostics, Fujirebio, Japan) as instructed by the manufacturer.

As a criterion for the clinical evaluation, the severity of stroke was assessed by NIHSS at baseline (NIHSS 0) and 48 hours after admission (NIHSS 1) by a trained neurology resident who was blinded to the study. Patient's long-time outcome was assessed using the BI with a structured follow-up telephone interview three months after hospital discharge by a trained nurse who was kept blinded to the study groups.

### 2.5. Definition

The NIHSS is a standard stroke assessment scale and measures neurologic impairment using 15 items. NIHSS categorizes stroke as mild (scores 1-4), moderate (scores 5-15), moderate to severe (scores 16-20), and severe (scores higher than 20) [20].

The BI is the standard scale used to measure performance in daily living activities. The BI measures 10 basic aspects of self-care and physical dependency. A normal score is 20, and lower scores indicate an increasing disability. A BI higher than 12 corresponds to assisted independence, and a BI lower than 8 corresponds to severe dependency [21].

NSE is one of the biomarkers of the brain and vascular injury. The reported range in 95% of the healthy subject is <12.5 ng/mL [22]. The NSE level increases within 2-3 hours after onset of the stroke symptoms and then decrease until 12 hours, and the second increase is until day 5 [15].

### 2.6. Outcomes

The primary objective of this study was to evaluate the effect of high doses of vitamin D_3_ on the NSE level, as the biomarker of brain damage in patients with moderate ischemic stroke. The effects of this regimen on neurological functions, according to NIHSS and BI scores, were evaluated as secondary outcomes.

### 2.7. Sample Size

The sample size of the study was calculated with Minitab software using 2-sample*t*-test function considering type I error of 0.05 and power of 0.8. The NSE level was considered 5.03 ± 3.25 ng/mL in the intervention group and 10.04 ± 5.72 ng/mL in the control group [23]. The sample size was calculated as 17 in each group. Considering 20% lost to follow-up, we considered 20 patients in each group.

### 2.8. Statistical Analysis

All statistical analyses were performed, using SPSS for Windows (version 21.0; SPSS Inc., Chicago, IL, USA). Quantitative data were tested for normality of distributions by the Kolmogorov–Smirnov test. The data are presented as mean ± standard deviation (SD) or median (percentile, Q1, Q3) for normal and nonnormal distribution, respectively. Two groups were compared by unpaired Student's *t*-test and Mann–Whitney *U* test for normal and nonnormal distribution data, respectively. Qualitative data were analyzed by the chi-squared test. A *p* value of <0.05 was considered significant.

## 3. Results

### 3.1. Baseline Data

**Figure 1:**
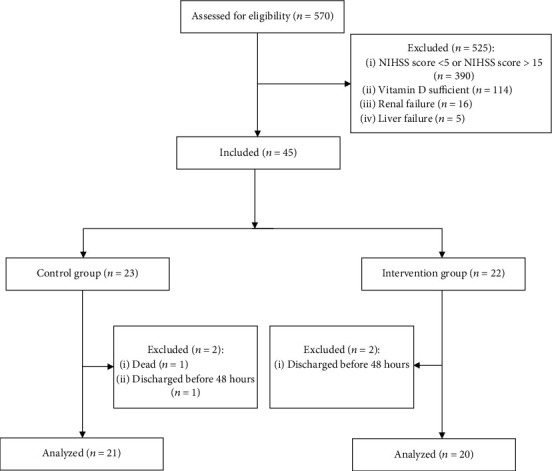
Consort chart of the study.

During the study period, 570 patients were assessed according to the eligibility criteria, and forty-five patients with moderate ischemic stroke were randomized into two groups of this study. Finally, forty-one patients completed the study, whereas 20 (48.7%) of them were in the intervention group (Figure 1). The baseline data of the two groups of the study are shown in Table 1. Except for the age, which was higher in the control group (*t* (28.64) = 2.145, *p* = 0.04), there was no statistically significant difference in other baseline characteristics between the two groups.

### 3.2. Serum NSE Levels

The median (Q1, Q3) of the serum NSE levels in the baseline (NSE 0) was 16.42 ng/mL (15.92, 18.01) and 16.04 ng/mL (15.67, 17.08) in the control and intervention groups, respectively. There was no statistically significant difference in comparison with NSE 0 between the two groups (*U*(*N*_Control group_ = 21, *N*_Intervention group_ = 19) = 161.0, *z* = −1.043, *p* = 0.29). Also, 48 hours after the intervention, there was no statistically significant difference in NSE 1 between the two arms of the study (6.74 ng/mL (5.93, 7.38) vs. 6.42 ng/mL (6.11, 9.87) in the control and intervention groups, respectively; *U*(*N*_Control group_ = 17, *N*_Intervention group_ = 16) = 129.0, *z* = −252, *p* = 0.80) (Table 2).

The decrement of serum NSE levels were -9.64 ng/mL (-11.31, -8.06) vs. -9.98 ng/mL (-11.60, -9.20) in the control and intervention arms of the study, which did not revealed a statistically significant difference (*U*(*N*_Control group_ = 17, *N*_Intervention group_ = 15) = 109.0, *z* = −0.699, *p* = 0.48) (Table 2).

### 3.3. Neurological Function Assessment Scales

As shown in Table 2, the baseline NIHSS (NIHSS 0) did not show statistically significant difference between the two groups (median (Q1, Q3) was 8 [8, 9] and 8 [6, 8] in the control and intervention groups, respectively; *U*(*N*_Control group_ = 21, *N*_Intervention group_ = 20) = 145.5, *z* = −1.760, *p* = 0.07), but 48 hours after the intervention, NIHSS 1 was significantly lower in the intervention arm of the study (median (Q1, Q3) was 8 [7, 8] and 6.5 (5.5, 7) in the control and intervention groups, respectively; *U*(*N*_Control group_ = 21, *N*_Intervention group_ = 20) = 110.5, *z* = −2.666, *p* = 0.008) (Table 2).

All patients were monitored after three months from hospital discharge, and the BI was calculated and recorded by a trained nurse. The analysis showed that the BI was significantly higher in the intervention group of the study (median (Q1, Q3) was 8 (7.5, 8.5) and 9 [8, 9] in the control and intervention groups, respectively; *U*(*N*_Control group_ = 16, *N*_Intervention group_ = 17) = 81, *z* = −2.098, *p* = 0.03).

## 4. Discussion

The current study revealed that a single high-dose vitamin D_3_ injection could significantly improve the neurological function of patients with moderate ischemic stroke, evaluated by the NIHSS and BI as the standard tools for evaluation of neurological function in patients with stroke. This effect was not detected on NSE as a biomarker of neurological damage.

Several observational studies showed worsening stroke severity, based on the NIHSS, and poor poststroke functional outcomes, assessed by the modified Rankin Scale (mRS) or BI scores at the discharge and/or 3-month poststroke, in patients with vitamin D deficiency [24–27].

Most studies examined the low doses of vitamin D on stroke-related comorbidities and complications such as neuromuscular disorders, osteoporosis, falls, and fractures in ischemic stroke patients [11, 28–30]. Improved muscle strength, reduction in falls, and decreased risk of hip fractures were obtained in poststroke patients with the intake of 700-1000 IU/day of vitamin D [28, 29].

The use of IM injection of single high-dose vitamin D in patients with ischemic stroke can be an appropriate treatment option in patients with poor compliance to daily oral vitamin D supplementation and could increase serum 25-OH vitamin D level rapidly and safely [16]. A few studies have examined the effect of high doses of vitamin D on the stroke severity and functional outcome scales of patients with ischemic stroke [17, 18, 31]. In accordance with our findings, Narasimhan et al. and Sari et al. revealed that a single dose, IM injection of 300000 to 600000 IU of vitamin D could improve functional outcomes of patients with ischemic stroke including Scandinavian Stroke Scale (SSS), mRS, NIHSS, and BI besides their balance [17, 18], whereas Rezaei et al. showed a single dose of 300000 IU IM vitamin D had no favorable effects on NIHSS score [31].

To the best of our knowledge, the exact mechanism of vitamin D in the improvement of neurological function of patients with ischemic stroke is not completely understood. NSE is a biomarker for acute ischemic stroke, and it is proven that serum concentration of NSE is correlated with the volume of infarcted tissue [13, 15]. A study that evaluated a high dose of vitamin D on the serum biomarkers such as NSE has not been conducted, but in the current study, we evaluated the NSE serum levels in moderated ischemic stroke patients but did not find any significant difference between the two arms of the study regarding this biomarker.

Serum NSE level increases during the first 24 hours after the stroke. After a decrease in its level, again, the NSE level rises on the 5^th^ day of poststroke [15]. The limitation of this study was that we only evaluated serum NSE levels at the baseline (first 24 hours) and 48 hours after the stroke. Generally, the patients with moderate ischemic stress discharge from the hospital before 5 days, so we were not able to take a blood sample on the 5^th^ day of the poststroke. So, we could not follow the second peak of NSE. For the future studies, we recommended the sequence evaluation of serum NSE levels until day 5 after the onset of the ischemic stroke.

## 5. Conclusion

In the conclusion, administration of a single 600000 IU of vitamin D_3_ could have neuroprotective effects in patients with moderate ischemic stroke, according to its significant positive effects on functional clinical outcomes (NIHSS and BI), but this effect on the biomarker related to neural damage (NSE) was not significant.

## Figures and Tables

**Table 1:** Baseline data of two groups of the study.

		Intervention group	Control group	*p* value^a^
Age (year) (mean ± SD)		60.05 ± 7.75	64.24 ± 4.12	0.04
Sex (*N* (%))	Male	14	14	0.81
	Female	6	7	
25-OH vitamin D (ng/mL) (mean ± SD)		23.20 ± 4.15	23.14 ± 4.29	0.96
Hospital length of stay (day) (mean ± SD)		2.05 ± 0.75	1.95 ± 0.65	0.80

^a^Unpaired Student's *t*-test and chi-squared test based on the data.

**Table 2:** Assessment data during study days.

		Intervention group	Control group	*p* value^a^
NSE (ng/mL) (median (Q1, Q3))	Baseline	16.04 (15.67, 17.08)	16.42 (15.92, 18.01)	0.29
	48 h after intervention	6.42 (6.11, 9.87)	6.74 (5.93, 7.38)	0.80
	Differences	-9.98 (-11.60, -9.20)	-9.64 (-11.31, -8.06)	0.48

NIHSS (median (Q1, Q3))	Baseline	8 (6, 8)	8 (8, 9)	0.07
	48 h after intervention	6.5 (5.5, 7)	8 (7, 8)	0.008

^a^Mann–Whitney *U* test.

## Data Availability

The data that support the findings of this study are available upon a reasonable request from the corresponding author, RH. The data are not publicly available due to the containing information that could compromise the privacy of research participants.
